# Cytokine characteristic of cerebrospinal fluid from children with enteroviral meningitis compared to bacterial meningitis

**DOI:** 10.1002/jcla.23198

**Published:** 2020-01-08

**Authors:** Jialu Xu, Jingjing Jiang, Yi Zhang, Wei Li

**Affiliations:** ^1^ Department of Neurology The Children’s Hospital Zhejiang University School of Medicine National Clinical Research Center for Child Health Hangzhou China; ^2^ Department of Clinical Laboratory Children’s Hospital Zhejiang University School of Medicine National Clinical Research Center for Child Health Hangzhou China

**Keywords:** bacterial meningitis, cytokines, enteroviral meningitis

## Abstract

**Background:**

Enteroviruses are the most common etiological agent for viral encephalitis, but it is uncertain whether the cytokines have the ability to differentiate enteroviral meningitis (EVM) from bacterial meningitis (BM).

**Methods:**

A retrospective study was performed at the Children's Hospital, Zhejiang University School of Medicine from August 2016 and August 2019. CSF and/or blood specimens were collected for microbiological culture, viruses, and cytokine detection.

**Results:**

Forty‐three patients were confirmed with meningitis, 27 patients with EVM, and 16 with BM. Children with EVM were older compared with BM and Control group (*P* < .001). The most common presenting symptom in children with EVM was fever (96.3%) followed by headache (88.9%) and vomiting (66.7%). The occurrence of seizure was lower in both EVM and BM groups (*P* < .001). Serum IL‐6 and serum IL‐10 were lower in EVM group than BM (*P* = .02) and control group (IL‐6, *P* = .01; IL‐10, *P* < .001). IL‐6, IL‐10, and IFN‐γ levels showed obviously increase in CSF (*P* < .001, respectively) in EVM group, while only IL‐6 increased in CSF (*P* < .001) in BM group. CSF concentrations of cytokines IL‐6, IL‐10, TNF, and IFN‐γ in children with EVM and BM were both higher than Control group (*P* < .001). But compared EVM group to BM group, CSF IL‐2 (*P* = .13), IL‐6 (*P* = .37), IL‐10 (*P* = .98), TNF (*P* = .54), and IFN‐γ (*P* = .53) showed no difference between two groups.

**Conclusions:**

CSF cytokines elevated in both virus and bacterial meningitis, while serum elevation only occurred in bacterial infection. Still, we could not distinguish enteroviral meningitis from bacterial meningitis with the parameters of CSF cytokines IL‐2, IL‐6, IL‐10, TNF, and IFN‐γ.

## 1 INTRODUCTION

Enteroviruses (EVs) are the most common etiological agent for viral meningitis in China.^1^ EVs are small, single‐stranded, positive sense RNA viruses including 11 species of human EV (Enterovirus A‐L and Rhinovirus A‐C).^2^ Enteroviral meningitis (EVM) are predominantly reported among children and always present as a milder clinical illness than other infectious causes 3 which only need supportive therapy. On the other hand, bacterial meningitis (BM) is still associated with a high morbidity and mortality rate.^4^ Appropriate antibiotics treatment is essential to optimize outcomes. In the previous study, cytokines were regarded as important roles in meningeal inflammation in patients with CNS infection. Cerebrospinal fluid (CSF) cytokines were reported as useful markers to distinguish viral and bacterial meningitis.^5^, ^6^ In this study, we investigated the cytokine profiles in the CSF and serum of children with EVM and BM, as well as with febrile seizures as a control group, to verify whether cytokines could be a useful indicator to distinguish EVM and BM.

## 2 PATIENTS AND METHODS

### 2.1 Study population

The study was designed as a retrospective study using the database of children older than 1 month of age at the Children's Hospital, Zhejiang University School of Medicine between August 2016 and August 2019. The study was approved with the ethics committee of Children's Hospital, Zhejiang University School of Medicine (2019‐IRB‐091). We categorized patients into three groups: (a) EVM group: Patients with EVM were confirmed by the presence of pleocytosis and detection of enteroviral nucleic acid detection in CSF using polymerase chain reaction (PCR).^7^ Patients who received immunomodulators or had other viral such as Epstein‐Barr virus (EBV), herpes simplex virus (HSV), or bacterial infections, or immune disorders were excluded; (b) BM group: Patients with bacterial meningitis were diagnosed as having CSF white blood cell counts >50 × 10^6^ cells/L with predominant neutrophils, CSF blood glucose ratio <0.5, CSF protein >50 mg/dL and positive Gram stain, and/or CSF culture; and (c) Control group: children with febrile seizures who underwent lumbar puncture and had normal CSF.

### 2.2 Pathogen detection

CSF specimens and blood samples were taken for microbiological culture. Samples were injected into children's blood culture bottles (BACTEC FX400, BD) for enrichment culture. Samples were then inoculated to appropriate medium according to smear results after culture positive. MALDI‐TOF (BRUKER) was used for bacterial identification and VITEK 2 compact (Biomerieux) for bacterial drug sensitivity. CLSI M100 S28/S29 were used as drug‐sensitive points.

The detection for viruses in CSF was following the manufacturer's instructions. CSF samples were collected from the children with viral meningitis. For the detection of EVs, a total of 200 µL CSF were taken for virus RNA extraction by using magnetic beads following nucleic acid automatic extraction instrument (Zhi‐jiang Company). The detection of EVs in ABI Step one plus system was performed by using commercial one‐step real‐time RT‐PCR assay kit (Zhi‐jiang company).^8^ The real‐time RT‐PCR was conducted under these conditions: 15 minutes at 50°C, 5 minutes at 95°C, and then followed by 40 cycles of 15 seconds at 94°C and 45 seconds at 55°C.

For the detection of nucleic acid of EB and HSV, a total of 50 µL CSF were mixed with 50 µL of DNA extraction solution. The mixture was boiled for 10 minutes, after which it was centrifuged at 4°C. Finally, a volume of 5 µL of the supernatant and 45 µL of PCR mix (Da'an Gene Co., Ltd.) were utilized to perform real‐time PCR using the Applied Biosystems 7500 real‐time PCR system (Applied Biosystems) according to the following protocol: 93°C for 2 minutes, 10 cycles of 93°C for 45 seconds, and 55°C for 60 seconds, followed by 30 cycles of 93°C for 30 seconds and 55°C for 45 seconds. Samples with CT value <35.0 were identified positive.

### 2.3 Cytokine detection

Serum and CSF concentration of cytokines interleukin (IL)‐2, IL‐6, IL‐10, tumor necrosis factor (TNF), and interferon (IFN)‐γ were quantitatively determined by the CBA kit–BDTM CBA Human Th1/Th2 Cytokine Kit II (BD Biosciences), according to the manufacturer's instructions. The minimal and maximum limits of detection for all six cytokines were 1.0 and 5000 pg/mL, respectively.

### 2.4 Statistical analysis

Data were analyzed using SPSS 18.0 statistical software and presented as median. We used Pearson's chi‐squared test or Fisher's exact test for categorical data and non‐parametric test. Mann‐Whitney *U* test, Kruskal‐Wallis test, and Nemenyi test were used for continuous data to determine significance. A two‐tailed *P* value of < .05 (*P* < .05) was considered to be statistically significant.

## 3 RESULT

### 3.1 Comparison of demographic and clinical features in EVM, BM, and Control groups

Of 43 patients with meningitis, 27 patients (eight girls, 19 boys) had EVM and 16 (five girls, 11 boys,) had BM. Children with EVM, that had median age of 7.2 (0.2‐14.6) years old, were older compared with BM group (median age 1.8 years old, range from 0.1 to 12.4) and Control group (median age 0.3 years old, range from 0.4 to 5.4; *P* < .001). The most common presenting symptom in children with EVM was fever (96.3%) followed by headache (88.9%) and vomiting (66.7%). And the occurrence of headache and vomiting was especially higher in EVM group compared with BM group and Control group (*P* < .05). The occurrence of seizure was lower in both EVM and BM groups (*P* < .001). We did lumber puncture at the time of clinical suspicion of meningitis, and the timing of lumber puncture from onset of symptoms had no difference among these three groups (*P* = .72). The demographic and clinical features of EVM, BM, and Control groups are presented in Table 1.

**Table 1:** The demographic and clinical features in EVM, BM, and Control groups

Clinical syndrome (n)	EVM (27)	BM (16)	Control (29)	*P*	EVM vs BM	EVM vs Control	BM vs Control
Gender, female/male	8/19	5/11	12/17	.63			
Age, median (range), y	7.2 (0.2‐14.6)	0.3 (0.1‐12.4)	1.8 (0.4‐5.4)	<.001	<0.001	<0.001	0.22
Fever (%)	26 (96.3)	16 (100)	29 (100)	.60			
Headache (%)	24 (88.9)	1 (6.3)	0	<.001	<0.001	<0.001	0.37
Vomiting (%)	18 (66.7)	5 (31.2)	2 (6.7)	<.001	0.04	<0.001	0.08
Seizure (%)	2 (7.4)	4 (25)	29 (100)	<.001	0.17	<0.001	<0.001
Timing of lumber puncture from onset of symptoms, median (range), d	2 (1‐10)	2.5 (1‐8)	2 (1‐8)	.72			

### 3.2 Cytokine expression of EVM, BM, and Control groups

The serum cytokines data in EVM, BM, and Control groups are presented in Table 2. Serum IL‐2, TNF, and IFN‐γ showed no differences among these three groups. The median level (pg/mL) of serum IL‐6 in EVM group was 15.15 (range from 1.20 to 707.60) pg/mL, which was obviously lower than BM (median 102.90, range from 2.80 to 3948.40, *P* = .02) and Control (median 116.10, range from 2.90 to 3726.30, *P* = .01) groups. Meanwhile, the median level (pg/mL) of serum IL‐10 (median 3.70, range from 1.50 to 21.80) in EVM group also appeared lower than BM (median 9.00, range from 1.90 to 137.00, *P* = .02) and Control (median 15.70, range from 1.00 to 281.90, *P* < .001) groups.

**Table 2:** The serum cytokines data in EVM, BM, and Control groups

Cytokine (pg/mL)	EVM	BM	Control	*P*	EVM vs BM	EVM vs Control	BM vs Control
Serum IL‐2	2.10 (1.00‐3.80)	2.50 (1.00‐5.10)	1.70 (1.00‐12.10)	.11			
Serum IL‐6	15.15 (1.20‐707.60)	102.90 (2.80‐3948.40)	116.10 (2.90‐3726.30)	.003	0.02	0.01	1.0
Serum IL‐10	3.70 (1.50‐21.80)	9.00 (1.90‐137.00)	15.70 (1.00‐281.90)	<.001	0.02	<0.001	0.67
Serum TNF	2.25 (1.00‐8.70)	2.50 (1.00‐9.50)	3.8 (1.00‐90.70)	.73			
Serum IFN‐γ	2.35 (1.00‐11.80)	4.30 (1.00‐107.00)	1.90 (1.00‐334.9)	.10			

**Figure 1:**
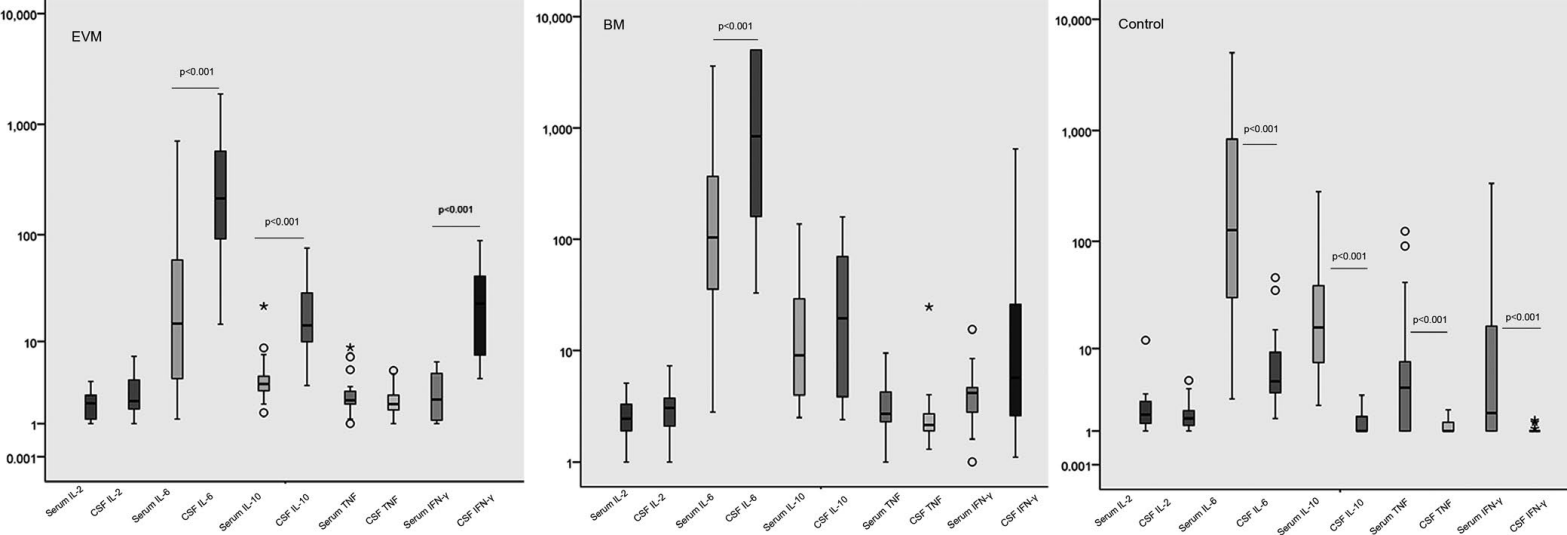
The serum and CSF cytokines data in EVM, BM, and Control groups

The median levels (pg/mL) of CSF cytokines IL‐2, IL‐6, IL‐10, TNF, and IFN‐γ in children with EVM were 1.95 (1.62‐7.10), 222.50 (96.65‐1872.40), 13.40 (8.7‐75.70), 2.00 (1.63‐5.00), and 20.10 (7.40‐88.10), respectively. Compared with serum levels, IL‐6, IL‐10, and IFN‐γ all showed obviously increase in CSF (*P* < .001, respectively). CSF cytokines of IL‐2, IL‐6, IL‐10, TNF, and IFN‐γ in children with BM were 3.15 (2.18‐7.50), 848.85 (165.83‐5000.00), 19.6 (3.93‐310.00), 2.15 (1.90‐68.60), and 4.80 (2.60‐647.90), respectively. Compared with serum levels, only IL‐6 showed obviously increase in CSF (*P* < .001). And in Control group, CSF cytokines of IL‐2, IL‐6, IL‐10, TNF, and IFN‐γ were 1.60 (1.20‐4.70), 4.60 (3.40‐46.80), 1.10 (1.00‐3.20), 1.00 (1.00‐2.10), and 1.00 (1.00‐3.00), respectively. Compared with serum levels, IL‐6, IL‐10, TNF, and IFN‐γ all decreased in CSF (*P* < .001, respectively). The data were shown in Figure 1.

Comparing of CSF cytokines data in EVM, BM, and Control groups was presented in Table 3. All these cytokines had significant differences among three groups (*P* < .001). Compared EVM group with Control group, CSF IL‐6, IL‐10, TNF, and IFN‐γ were higher in EVM group (*P* < .001, respectively) except CSF IL‐2 (*P* = .07). Compared BM group with Control group, CSF IL‐2, IL‐6, IL‐10, TNF, and IFN‐γ were all higher in BM group (*P* < .001, respectively). But compared EVM group with BM group, CSF IL‐2 (*P* = .13), IL‐6 (*P* = .37), IL‐10 (*P* = .98), TNF (*P* = .54), and IFN‐γ (*P* = .53) all showed no difference between two groups.

**Table 3:** The CSF cytokines data in EVM, BM, and Control groups

Cytokine (pg/mL)	EVM	BM	Control	*P*	EVM vs BM	EVM vs Control	BM vs Control
CSF IL‐2	1.95 (1.62‐7.10)	3.15 (2.18‐7.50)	1.60 (1.20‐4.70)	<.001	0.13	0.07	<0.001
CSF IL‐6	222.50 (96.65‐1872.40)	848.85 (165.83‐5000.00)	4.60 (3.40‐46.80)	<.001	0.37	<0.001	<0.001
CSF IL‐10	13.40 (8.7‐75.70)	19.6 (3.93‐310.00)	1.10 (1.00‐3.20)	<.001	0.98	<0.001	<0.001
CSF TNF	2.00 (1.63‐5.00)	2.15 (1.90‐68.60)	1.00 (1.00‐2.10)	<.001	0.54	<0.001	<0.001
CSF IFN‐γ	20.10 (7.40‐88.10)	4.80 (2.60‐647.90)	1.00 (1.00‐3.00)	<.001	0.53	<0.001	<0.001

## 4 DISCUSSION

With the popularization of bacterial conjugate vaccines, the epidemiology of childhood meningitis has dramatically changed and viral causes are increasingly predominant.^9^ EVs are the most common etiological agent for viral meningitis. Compared children with EVM with children with BM, they had no gender difference. But children with EVM had the median age of 7.2 (0.2‐14.6) years old which was mainly preschool or school age children, while BM mainly occurred in children under 1 year old. EVM usually had a conjunction with encephalitis resulting in a “meningoencephalitis”.^10^, ^11^ The predominant symptom of EVM in our study was fever (96.3%), headache (88.9%), and vomiting (66.7%). The incidence of seizure was only 7.4%, as Berardi A et al reported the incidence of seizure was low both in infants <90 days of age and children older than 90 days of age.^12^ Meanwhile in adult, the incidence of seizure was also low to 4%.^13^ It seems enterovirus meningoencephalitis is associated with low seizure occurrence.

Serum IL‐6 and serum IL‐10 were obviously lower in patients with EVM that seemed EVM could not trigger the involvement of the peripheral immune system. In CSF, IL‐6, IL‐10, and IFN‐γ showed rapidly increase in EVM group while cytokines sharply declined in control group without meningeal infection. The elevation of CSF pro‐inflammatory cytokine IL‐6, TNF, and IFN‐γ and anti‐inflammatory cytokine IL‐10 was approved in EVM,^14^, ^15^, ^16^ that meant meningeal inflammation could trigger a unified pro‐ and anti‐inflammatory response in viral infections.^17^, ^18^, ^19^, ^20^ Cytokine profiling could contribute to localize the site of immune activation in viral infections. Even cytokines seemed to have the ability to distinguish enterovirus infection from other viruses. Enterovirus meningoencephalitis group showed higher level of CSF IL‐6, TNF, and IL‐17 when compared to arbovirus, lentivirus, and herpes groups.^21^ While compared to patients with human parechovirus meningitis, CSF levels of the majority cytokines were still significantly higher in patients with EVM.^22^

In BM group, compared with serum, only IL‐6 elevated in CSF. IL‐6 played to amplify an acute inflammation as well as to contribute to the transition into the chronic phase of inflammation.^23^ Previous study reported IL‐6 as well as IL‐1β, IL‐8, TNF‐α, and GM‐CSF to be useful markers to distinguish bacterial and viral meningitis.^5^, ^6^, ^24^ But in our study, CSF levels of IL‐2, IL‐6, IL‐10, TNF, and IFN‐γ all showed no difference between EVM and BM groups. Though prominent CSF IFN‐γ elevation was found in EVM group,^25^ IFN‐γ was still not a sensitive indicator to distinguish EVE from BM.^26^ Also, we interpreted the involvement of the peripheral immune system in bacterial meningitis, while CSF and serum concentrations of the cytokines were not shown to be correlated under enterovirus infection.^27^

## 5 CONCLUSION

We detected the elevation of CSF pro‐inflammatory and anti‐inflammation cytokines in meningeal inflammation with both enterovirus and bacterial infection, while serum elevation only occurred in bacterial infection. Thus, we could not distinguish enteroviral meningitis from bacterial meningitis with the parameters of CSF cytokines IL‐2, IL‐6, IL‐10, TNF, and IFN‐γ. More related cytokines need to be investigated under even larger samples.
